# Impact of Refutational Two-Sided Messages on Attitudes Toward Novel Vaccines Against Emerging Infectious Diseases During the COVID-19 Pandemic

**DOI:** 10.3389/fpubh.2022.775486

**Published:** 2022-02-11

**Authors:** Hideo Okuno, Satoru Arai, Motoi Suzuki, Toshiko Kikkawa

**Affiliations:** ^1^Center for Surveillance, Immunization, and Epidemiologic Research, National Institute of Infectious Diseases, Tokyo, Japan; ^2^Division of Infection Control and Prevention, Osaka University Hospital, Osaka, Japan; ^3^Faculty of Business and Commerce, Keio University, Tokyo, Japan

**Keywords:** risk communication, vaccine hesitancy, refutational two-sided messages, inoculation theory, COVID-19, two-sided messages, anticipated regret

## Abstract

Two-sided messages that include two perspectives (i.e., risks and benefits) are more effective than one-sided messages that convey only one perspective (usually only the benefits). Refutational two-sided messages are effective for communicating risks regarding vaccines. To examine the effectiveness of refutational two-sided messages in risk communication regarding novel vaccines against emerging infectious diseases, we conducted the randomized controlled study based on a 3 × 3 × 2 mixed design (Intervention 1: vaccines against subcutaneous influenza, “novel severe infectious disease,” or intranasal influenza; intervention 2: one-sided, non-refutational two-sided, or refutational two-sided messages; two questionnaires) using a Japanese online panel. Participants completed questionnaires before and after receiving an attack message (negative information). We evaluated the impact of attack messages on the willingness to be vaccinated, and the anticipated regret of inaction (ARI). Among 1,184 participants, there was a significant difference in the willingness to be vaccinated among the message groups (*p* < 0.01). After receiving the attack message, willingness to be vaccinated decreased in the one-sided message group and increased in the non-refutational two-sided and refutational two-sided message groups. Additionally, ARI in the refutational two-sided message groups was significantly higher than in the one-sided groups (*p* = 0.03). In conclusion, two-sided messages are more effective than one-sided messages in terms of willingness to be vaccinated. Furthermore, the high ARI in the refutational two-sided message group indicated that refutational two-sided messages were more effective than one-sided messages for communicating the risks of vaccines, especially those against emerging infectious diseases.

## Introduction

The pace of vaccination against coronavirus disease 2019 (COVID-19) has been accelerating in many countries, to establish herd immunity. However, vaccine hesitancy among policy-makers and clinicians is a major obstacle to vaccination efforts (1). Japan is also facing this obstacle (2, 3). In Japan, the national program for human papillomavirus (HPV) vaccine was stopped because vaccine side effects caused controversies that led to a steep decline in vaccine coverage (<1% of Japanese population) and serious risks for unvaccinated women (4, 5). COVID-19 vaccine hesitancy may not be completely unreasonable considering the controversies surrounding vaccines such as the measles, mumps, and rubella vaccine or HPV vaccine (6).

Emphasis on the benefits of vaccines, without considering their side effects, is risky despite its short-term positive effects. This strategy may not work because people's attitudes toward the vaccine change if they encounter negative information regarding it (e.g., vaccine side effects). Additionally, the environment cannot be cleared of negative information regarding the vaccine (correct or incorrect) (7), and vaccines do have some risks for certain populations; people should have the right to decide whether they want to be vaccinated. The best strategy to gain the public's trust in a crisis like COVID-19 is to inform them of the positive and negative scientific evidence on vaccination.

The psychological inoculation theory states that beliefs called “cultural truisms” such as “the effects of penicillin have been of enormous benefit to mankind” are vulnerable to counterarguments (8). The mechanism of resistance to counterarguments can be explained using the analogy of medical inoculation. People will be motivated to defend their attitudes if they are already informed about possible arguments including mere forewarnings. People who have been informed of the possible arguments are likely to refute them and become resistant to negative information, a process called attitudinal inoculation. Therefore, people build immunity against future attacks.

Although this theory helps us understand the mechanism of resistance to persuasion, we must recognize that the mechanism is not identical between medical and psychological contexts. Compton pointed out that, in the medical context, the immune system is automatically motivated, while in the psychological (persuasion) context, cognitive affective systems are motivated by recognition of a threat (9).

The applicability of this theory to other fields has been explored (8, 10). In particular, its application to health-related issues (11), including vaccination (12), has been increasing.

Studies have demonstrated the superiority of two-sided messages related to vaccination over one-sided messages. One-sided messages present a single perspective, typically positive, whereas two-sided messages present arguments from both sides, i.e., both the risks and benefits. A two-sided message is more effective than a one-sided message because it increases the credibility of the message and functions as attitudinal inoculation, i.e., motivates people to “protect” their attitude. Refutational two-sided messages refute counterarguments and are more effective (13). Such messages have been used to tackle misinformation regarding the measles, mumps, and rubella vaccine (14).

For effective risk communication regarding the COVID-19 vaccine, the credibility of information on its safety and effectiveness is an important factor in the decision to get vaccinated, especially among those who are unsure about getting vaccinated (15). However, to the best of our knowledge, no study has evaluated whether refutational and non-refutational two-sided messages are effective in the case of vaccines for life-threatening diseases, such as COVID-19. The risks of vaccines should be communicated accurately because risk perception increases for unfamiliar interventions (16).

In the present study, we compared the effectiveness of refutational two-sided messages with one-sided and non-refutational two-sided messages, to understand how better to convince people regarding the benefits of COVID-19 vaccination.

## Materials and Methods

We conducted a randomized controlled study using a 3 × 3 × 2 mixed design (Intervention 1: vaccines against subcutaneous influenza, “novel severe infectious disease,” or intranasal influenza; intervention 2: one-sided, non-refutational two-sided, or refutational two-sided messages; two questionnaires) (Supplementary Figure 1). The first two interventions were between-subjects variables, and the third was a within-subjects variable. The study participants were recruited in December 2020 using an online panel provided by the NTTCom Online Marketing Solutions Corporation (Tokyo, Japan). During the study period, COVID-19 vaccination had started in the USA, and there was a paucity of information regarding COVID-19 vaccine side effects in Japan. “Novel severe infectious disease” was a fictitious disease, and intranasal influenza vaccine had not been introduced in Japan.

We recruited 1,184 participants with approximately equal representation of sex and age groups. Informed consent was obtained online (Supplementary Figure 1). This study obtained ethical approval from the institutional review board of the National Institute of Infectious Diseases of Japan.

For Intervention 1, participants were asked to imagine that they received an explanation for one of the following vaccines: subcutaneous influenza vaccine, intranasal influenza vaccine, or “novel severe infectious disease” vaccine before vaccination. The participants were informed that the “novel severe infectious disease” was fictitious.

Irrespective of their assigned groups in Intervention 1, participants were randomly assigned to one of three experimental conditions: one-sided, non-refutational two-sided, or refutational two-sided messages. Participants in the one-sided message group were given positive information (i.e., general information and data on effectiveness) about the vaccine that they were assigned in Intervention 1. Positive information about the subcutaneous and intranasal influenza vaccines was adapted from the content on the websites of the US Centers for Disease Control and Prevention, the Japanese National Institute of Infectious Disease, and the Ministry of Health, Labour, and Welfare (17, 18). Positive information for the “novel severe infectious disease” was the same as that for the subcutaneous influenza vaccine, except for the different names of the infectious disease and the vaccine.

Participants in the non-refutational two-sided message group received the following message in addition to the positive information: “However, vaccine side effects occur in a certain proportion of recipients, estimated to be 15–30%.” (counterarguments) Participants in the refutational two-sided message group received the following message in addition to the messages received by the non-refutational two-sided message group: “Although vaccine side effects may occur in a certain proportion of the recipients, most side effects are relatively mild and improve in a few days. To date, documented serious side effects have been very rare” (refutation). The type of refutation was “refutational-same,” in the sense that both the counterargument and refutation deal with the side effects of vaccination (10). After reading the messages, all participants received an attack message (negative information) about the vaccine side effects. Information regarding the side effects of the subcutaneous and intranasal influenza vaccines was adapted from the content on the websites of the US Centers for Disease Control and Prevention and the Japanese Ministry of Health, Labour, and Welfare (17, 18). Information regarding the side effects of the vaccine against the “novel severe infectious disease” was the same as that for the subcutaneous influenza vaccine, except for the different names of the infectious disease, and the vaccine. We also added that there were no data from Japan regarding the serious side effects of the intranasal influenza and “novel severe infectious disease” vaccines.

Participants filled in questionnaires before and after receiving the attack message. Participants were asked to rate on Likert-type scales [range: 1 (not at all) to 5 (extremely)]: their willingness to be vaccinated, anxiety regarding vaccine side effects, anticipated regret if they did not get vaccinated and developed an infection (anticipated regret of action, ARA), and anticipated regret if they were vaccinated and suffered from vaccine side effects (anticipated regret of inaction, ARI).

We analyzed the data to evaluate the impact of the attack message on willingness to be vaccinated, especially against the “novel severe infectious disease.” Differences among the groups were analyzed using three-way factorial analysis of variance (ANOVA) and Bonferroni correction. *P* < 0.05 were considered significant. All analyses were performed using SPSS Statistics software (version 25; IBM Corp., Armonk, NY, USA).

## Results

### Study Participants

The average age of the participants was 46.3 years (SD = 13.6; Table 1). More than half had graduated from graduate schools or universities (*n* = 652; 54.7%; Table 1). Before Intervention 1, participants were asked to rate their interest in various real vaccines using Likert-type scales. The percentage of participants who were extremely interested in the vaccine along with the average score of interest was highest for the COVID-19 vaccine (36.4%; average score of interest: 3.83), followed by the subcutaneous influenza vaccine (19.9%; 3.17), and intranasal influenza vaccine (9.7%; 2.75; *p* < 0.01; Table 1). Regarding vaccine knowledge, the average knowledge scores were relatively low for unfamiliar vaccines, i.e., the COVID-19 and intranasal influenza vaccines, compared to the subcutaneous influenza vaccine (1.83, 2.61, 2,88, respectively; *p* < 0.001).

**Table 1:** Characteristics and interest in the vaccines.

		**All participants**	
		***n* = 1,193**	**(%)**
Average age (years, ±SD)		46.3 (±13.6)	
Male		644	54.0
Education			
	High school/Junior college	519	43.5
	University	581	48.7
	Graduate school	71	6.0
Interest in the subcutaneous influenza vaccine			
	Extremely	238	19.9
	Not at all	199	16.7
	Average scores (±SD)^a^	3.17 (±1.366)	
Interest in the novel severe infectious disease vaccine			
	Extremely	427	36.4
	Not at all	104	8.7
	Average scores (±SD)^a^	3.83 (±1.218)	
Interest in the nasal influenza vaccine			
	Extremely	116	9.7
	Not at all	297	24.9
	Average scores (±SD)^a^	2.75 (±1.282)	

a *Average scores of Likert-type scales ranging from 1 (not at all) to 5 (extremely). SD, standard deviation*.

### Comparison Among the Vaccines

**Figure 1:**
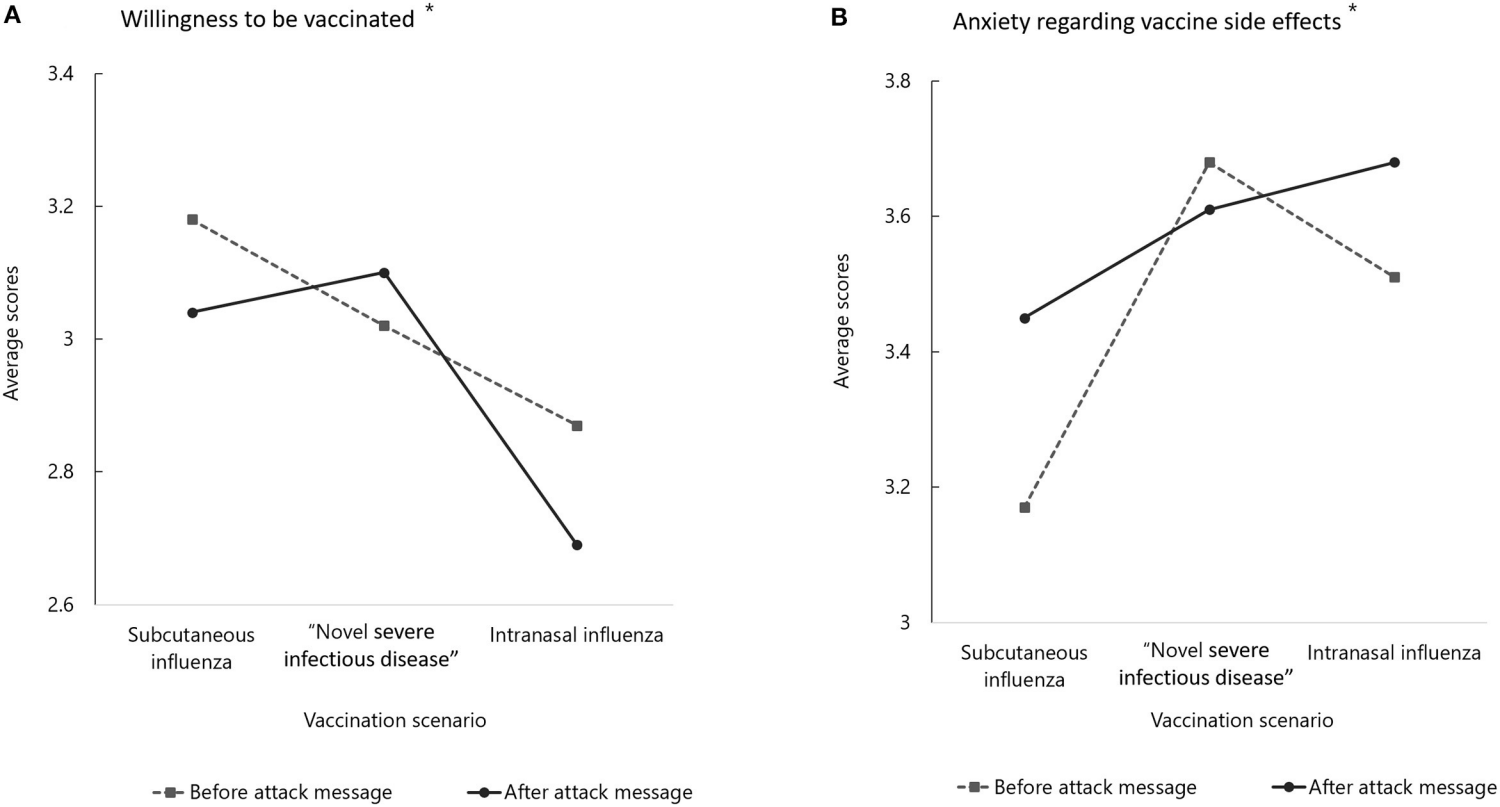
Evaluation of vaccination intention and anxiety regarding vaccine side effects between before and after the attack message among vaccination scenarios. *A significant interaction between the impact of the attack message and the vaccine scenarios was obtained (*p* < 0.01). “Novel severe infectious disease” is the scenario of the fictitious disease.

The attack message had a significant impact on willingness to be vaccinated [*F*_(2, 1, 184)_ = 14.204; *p* < 0.01]. Willingness to be vaccinated with the intranasal influenza vaccine was significantly lower compared to the other two vaccines. Willingness to be vaccinated with the subcutaneous and intranasal influenza vaccines decreased significantly after receiving the attack message. However, willingness to be vaccinated increased after receiving the attack message about the “novel severe infectious disease” vaccine (Figure 1A). The attack message significantly increased anxiety regarding vaccine side effects [*F*_(2, 1, 184)_ = 14.483; *p* < 0.01). Participants in the intranasal influenza vaccine group had significantly higher levels of anxiety than those in the other two groups (Figure 1B). However, in the “novel severe infectious disease” group, anxiety decreased after receiving the attack message (Figure 1B).

There was no significant impact of the attack message on the ARI (Supplementary Table 1). However, the ARI differed significantly between the vaccine groups [*F*_(2, 1, 184)_ = 37.966; *p* < 0.01]. ARI was higher in the “novel severe infectious disease” group than in the other groups. There was a significant interaction between the attack message and ARA. There were significant differences in the impact of negative interaction on the ARA among the groups [*F*_(1, 1, 184)_ = 16.032; *p* < 0.01]. There was a decrease in ARA in the “novel severe infectious disease” and intranasal influenza vaccine groups after receiving the attack message. However, the ARA in the subcutaneous influenza group increased after receiving the attack message (Supplementary Table 1).

### Evaluation of the Impact of the Message in the Novel Vaccine Group

**Figure 2:**
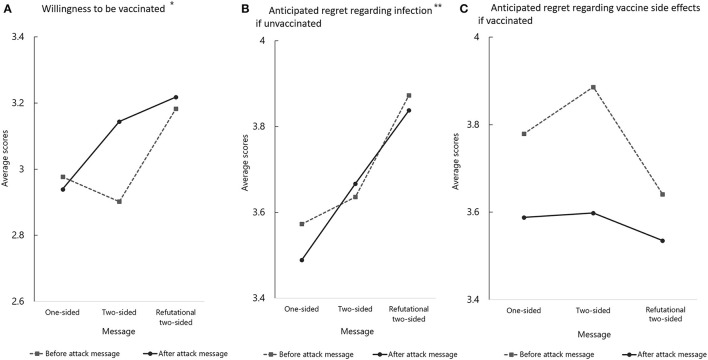
Evaluation of vaccination intention and anxiety regarding vaccine side effects between before and after the attack message in the fictitious infectious-disease scenario. *A significant interaction between the impact of the attack message and the message groups was obtained (*p* < 0.01). **Pairwise comparisons using Bonferroni correction revealed a significant difference between the one-sided and refutational two-sided message groups (*p* = 0.03).

In the “novel severe infectious disease” group, there was a significant difference between the message types in terms of willingness to be vaccinated [*F*_(2, 402)_ = 5.572; *p* < 0.01]. Willingness to be vaccinated was significantly lower in the one-sided message group than in the other two message groups (Figure 2A). Additionally, willingness to be vaccinated decreased in the one-sided message group after receiving the attack message. However, in the non-refutational two-sided and refutational two-sided message groups, willingness increased after receiving the attack message. There was a significant interaction between the impact of the attack message and the message groups in terms of anxiety regarding side effects (Supplementary Table 2). After receiving the attack message, anxiety increased in the one-sided message group and decreased in the non-refutational two-sided and refutational two-sided message groups.

ARI was highest in the refutational two-sided message group. Pairwise comparisons using Bonferroni correction revealed a significant difference between the one-sided and refutational two-sided message groups (*p* = 0.03; Figure 2B). ARA increased significantly after receiving the attack message [*F*_(1, 402)_ = 21.977; *p* < 0.01]; however, no significant differences were noted between the three message groups [*F*_(2, 402)_ = 0.84; *p* = 0.20; Figure 2C].

## Discussion

In this study, the impact of the attack message (negative information) differed significantly among the vaccine groups. In the “novel severe infectious disease” group, participants were more willing to be vaccinated and had lower anxiety regarding vaccine side effects after receiving the attack message (i.e., information about vaccine side effects) compared to the other two vaccine groups. Therefore, providing information regarding vaccine side effects does not necessarily induce reluctance to be vaccinated.

Participants in the refutational two-sided message group rated ARI higher for the “novel severe infectious disease” vaccine compared to the other vaccines. Because most people are risk-averse, they are motivated to avoid regret in the future (loss aversion). Anticipated regret is an important factor in risk perception and vaccine uptake (19–21). Reiter et al. reported that ARI led to greater willingness to receive the HPV vaccine (22). The higher ARI among participants of the refutational two-sided message group implies that these messages were more effective than one-sided messages in terms of risk communication, especially for a novel infectious disease. There were no significant differences among the message groups in terms of ARA. Therefore, informing individuals of the side effects of a new vaccine against a novel infectious disease does not necessarily lead to a negative attitude toward receiving the vaccine.

Because participants were unfamiliar with the arguments in support of the vaccination for the novel infectious disease, participants were not able to refute the arguments by themselves. Therefore, the refutational messages provided to the study participants informed them regarding the arguments and counter-arguments. These messages were useful for “inoculating” the patients against the attack message regarding vaccination. Their willingness and ARI increased after receiving the attack message in the form of refutational two-sided messages, indicating the effectiveness of refutation. After receiving the attack message, anxiety about vaccine side effects increased in the one-sided message group, implying that the participants in this group could not generate refutation arguments or defend their attitude against attacks. This could have implications for combatting misinformation and conspiracy theories about COVID-19, as Compton et al. suggested (23).

We used a fictitious name for an infectious disease to increase the generalizability of our results. The results of this study may be applied to new vaccines for diseases other than COVID-19, because we did not specify that the “novel severe infectious disease” was COVID-19. Our results could help policy-makers and medical experts to convince people to get vaccinated, i.e., by using refutational two-sided messages before they develop their own attitudes. When new vaccines are introduced, policy-makers may be inclined to emphasize the benefit of vaccines, to establish herd immunity, and to communicate paternalistically. However, this strategy is risky because contradictory evidence will eventually appear. As Ivanov and Parker pointed out (24), “inoculation-based messages are well-suited to assist the efforts of civic leaders to convince the public to accept the forthcoming coronavirus vaccine.” Our results reinforce their contention.

The relatively low willingness to be vaccinated with the intranasal influenza vaccine may be interpreted as follows. In Japan, subcutaneous influenza vaccine is commonly used, and people are familiar with the use of this vaccine. Therefore, the Japanese population are not aware of the practical benefits of the intranasal vaccine. Participants were less interested in the intranasal influenza vaccine compared to the COVID-19 or subcutaneous influenza vaccines (Table 1). The study participants were not willing to receive an unknown vaccine, especially when an alternative was available. Although the numbers of studies on the research and development of intranasal influenza vaccines have been increasing (25), further studies on risk communication are required, especially when a new intranasal influenza vaccine is introduced.

There are several limitations to this study. First, we used a scenario based on a fictitious disease because of ethical considerations. Therefore, we did not directly evaluate the effectiveness of refutational and non-refutational two-sided messages for COVID-19 vaccines. However, as described previously, we consider this a strength of this study rather than a limitation. Participants in this study were extremely interested in COVID-19 vaccines (Table 1). Therefore, it is possible that the participants assumed COVID-19 to be the “novel severe infectious disease.” To confirm or refute this, further studies are required that directly evaluate the effects of different types of messages on attitudes toward COVID-19 vaccines. Second, we did not directly measure threat. That is, we did not establish the threat level using conventional measures (12). As Compton and Pfau stated (26), “inoculation is impossible without threat.” Participants had little knowledge about vaccinations except for subcutaneous influenza vaccines when the study was conducted. In other words, they were in a “germ-free” situation, where any counterargument could be a threat. Future studies are necessary to confirm the validity of our interpretation. Third, we recruited participants using a Japanese online panel. Although two-sided messages (with or without refutation) have been reported to be superior to one-sided messages in studies conducted in many countries, attitudinal differences toward vaccination depend on local cultures and may reduce the effectiveness of these messages. Japanese society is characterized as privileging masculinity and focused on avoiding uncertainty as well as on long-term orientation. Therefore, attitudes toward vaccination may be more positive in Japan, as compared to other countries with different characteristics (27). In addition, anti-COVID-19 vaccination attitudes and conspiracy theories have not gained traction in Japan compared to other countries where these issues are a matter of serious concern (24, 28). Studies of the effects of the messages based on cultural differences will allow us to tailor messages to specific cultures (29). Finally, we demonstrated the short-term effects of refutational two-sided messages using an online survey. We did not evaluate the duration of the effects of these messages. Although the inoculation theory suggests that attitudinal immunity will last a long time (8), and that immunity provides umbrella protection against new arguments, generalizing the current results should be done with caution, since the pandemic is still ongoing and new arguments for and against vaccinations have been increasing (30).

## Conclusions

Our results are in agreement with those of previous studies, which demonstrated that refutational two-sided messages are effective for vaccine communication, especially for novel infectious diseases. To the best of our knowledge, this is the first study to show the effectiveness of refutational two-sided messages for risk communication for new vaccines introduced during the COVID-19 pandemic. Communicating the risks and benefits of vaccines is a fair, transparent, and effective strategy for vaccine communication.

Previous studies detected the superiority of refutational two-sided messages over one-sided messages for conventional vaccines. This study validated the results of previous studies using vaccines introduced during the COVID-19 pandemic.

## Data Availability Statement

The original contributions presented in the study are included in the article/Supplementary Material, further inquiries can be directed to the corresponding authors.

## Ethics Statement

The studies involving human participants were reviewed and approved by the National Institute of Infectious Diseases, Japan. The patients/participants provided their written informed consent to participate in this study.

## Author Contributions

HO and TK designed the study, analyzed data, and wrote the manuscript. HO, SA, and TK performed the study and collected data. SA and MS gave support and conceptual advice. All authors read and approved the final manuscript.

## Funding

This research was supported by AMED under Grant Number 20fk0108066h1003.

## Conflict of Interest

The authors declare that the research was conducted in the absence of any commercial or financial relationships that could be construed as a potential conflict of interest.

## Publisher's Note

All claims expressed in this article are solely those of the authors and do not necessarily represent those of their affiliated organizations, or those of the publisher, the editors and the reviewers. Any product that may be evaluated in this article, or claim that may be made by its manufacturer, is not guaranteed or endorsed by the publisher.

## Abbreviations

COVID-19: coronavirus disease 2019
HPV: human papillomavirus
PCV: pneumococcal conjugated vaccine
ARI: anticipated regret of inaction
ARA: anticipated regret of action
ANOVA: analysis of variance.
