# Supplementary figures and images for: Impact of Refutational Two-Sided Messages on Attitudes Toward Novel Vaccines Against Emerging Infectious Diseases During the COVID-19 Pandemic
Source: Front Public Health. 2022 Feb 11;10:775486. doi: 10.3389/fpubh.2022.775486 (PMC8873109; doi:10.3389/fpubh.2022.775486)

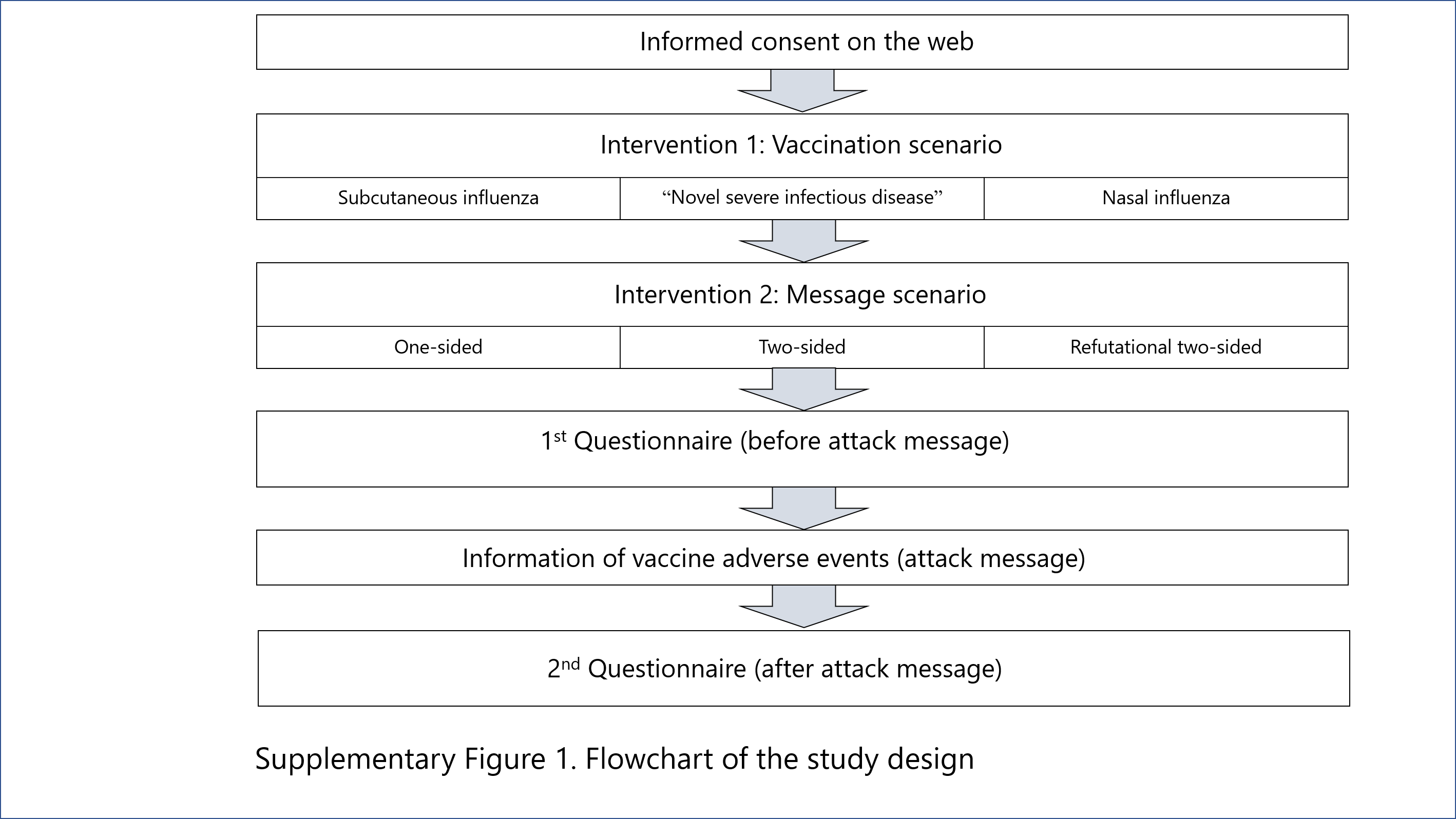

Supplement: Supplementary file 1 [file Image_1.TIF]
