# Supplementary material for: Impact of Refutational Two-Sided Messages on Attitudes Toward Novel Vaccines Against Emerging Infectious Diseases During the COVID-19 Pandemic
Source: Front Public Health. 2022 Feb 11;10:775486. doi: 10.3389/fpubh.2022.775486 (PMC8873109; doi:10.3389/fpubh.2022.775486)
Supplement: Supplementary file 2 [file Data_Sheet_1.docx]

| Supplementary Table 1. Average scores on Likert-type scales before and after the attack message among the three vaccine groups | | | | | | | | | | | |
| --- | --- | --- | --- | --- | --- | --- | --- | --- | --- | --- | --- |
|  |  |  | Vaccine groups | | | | | | Vaccine scenarios × attack message | | |
|  |  |  | Subcutaneous Flu | | "Novel severe infectious disease"^a^ | | Intranasal Flu | |  |  |  |
|  |  |  | n = 393 | (95% CI) | n = 405 | (95% CI) | n = 395 | (95% CI) | df | F value | p-value |
| Willingness to be vaccinated | |  |  |  |  |  |  |  |  |  |  |
|  | before attack message |  | 3.181 | 3.071–3.292 | 3.021 | 2.912–3.129 | 2.879 | 2.768–2.990 | 2 | 14.204 | < .001^b^ |
|  | after attack message |  | 3.032 | 2.922–3.142 | 3.100 | 2.922–3.209 | 2.702 | 2.592–2.812 |  |  |  |
| Anxiety regarding vaccine side effects | |  |  |  |  |  |  |  |  |  |  |
|  | before attack message |  | 3.173 | 3.076–3.269 | 3.682 | 3.587–3.777 | 3.502 | 3.405–3.599 | 2 | 12.483 | < .001^b^ |
|  | after attack message |  | 3.443 | 3.345–3.542 | 3.617 | 3.519–3.714 | 3.682 | 3.583–3.782 |  |  |  |
| Anticipated regret regarding infection, if unvaccinated | | | |  |  |  |  |  |  |  |  |
|  | before attack message |  | 3.13 | 3.016–3.244 | 3.700 | 3.593–3.807 | 3.12 | 3.002–3.238 | 2 | 0.987 | .373 |
|  | after attack message |  | 3.07 | 2.958–3.182 | 3.670 | 3.563–3.777 | 3.04 | 2.925–3.155 |  |  |  |
| Anticipated regret regarding vaccine side effects, if vaccinated | | | | | |  |  |  |  |  |  |
|  | before attack message |  | 3.594 | 3.491–3.698 | 3.769 | 3.667–3.871 | 3.848 | 3.744–3.952 | 2 | 12.489 | < .001^b^ |
|  | after attack message |  | 3.666 | 3.558–3.775 | 3.574 | 3.467–3.680 | 3.685 | 3.577–3.794 |  |  |  |
|  | a) "Novel severe infectious disease” is the scenario of a fictitious disease. | | | | |  |  |  |  |  |  |
|  | b) ANOVA revealed a significant interaction between the impact of the attack message and the message scenarios. | | | | | | |  |  |  |  |

CI: confidence interval; df: degrees of freedom; ANOVA: analysis of variance
