# Supplementary material for: Impact of Refutational Two-Sided Messages on Attitudes Toward Novel Vaccines Against Emerging Infectious Diseases During the COVID-19 Pandemic
Source: Front Public Health. 2022 Feb 11;10:775486. doi: 10.3389/fpubh.2022.775486 (PMC8873109; doi:10.3389/fpubh.2022.775486)
Supplement: Supplementary file 3 [file Data_Sheet_2.docx]

| Supplementary Table 2. Average scores on Likert-type scales before and after the attack message among the three message groups | | | | | | | | | | |  |
| --- | --- | --- | --- | --- | --- | --- | --- | --- | --- | --- | --- |
|  |  |  | Message scenario | | | | | | Message scenarios× the attack message | | |
|  |  |  | One-sided message | | Two-sided message | | Refutational two-sided message | |  |  |  |
|  |  |  | n = 131 | (95% CI) | n = 132 | (95% CI) | n = 142 | (95% CI) | df | F value | p-value |
| Willingness to be vaccinated | | | |  |  |  |  |  |  |  |  |
|  | before attack message |  | 2.977 | 2.789–3.166 | 2.902 | 2.714–3.089 | 3.183 | 3.002–3.234 | 2 | 5.572 | < .001^a^ |
|  | after attack message |  | 2.939 | 2.747–3.130 | 3.144 | 2.953–3.335 | 3.218 | 3.034–3.402 |  |  |  |
| Anxiety regarding vaccine side effects | | | |  |  |  |  |  |  |  |  |
|  | before attack message |  | 3.687 | 3.519–3.855 | 3.780 | 3.613–3.947 | 3.577 | 3.416–3.739 | 2 | 4.706 | .01^a^ |
|  | after attack message |  | 3.771 | 3.599–3.943 | 3.515 | 3.343–3.687 | 3.563 | 3.398–3.729 |  |  |  |
| Anticipated regret regarding infection, if unvaccinated | | | | |  |  |  |  |  |  |  |
|  | before attack message |  | 3.573 | 3.385–3.760 | 3.636 | 3.449–3.823 | 3.873 | 3.693–4.054 | 2 | 1.031 | .358 |
|  | after attack message |  | 3.489 | 3.302–3.675 | 3.667 | 3.481–3.853 | 3.838 | 3.659–4.017 |  |  |  |
| Anticipated regret regarding vaccine side effects, if vaccinated | | | | |  |  |  |  |  |  |  |
|  | before attack message |  | 3.779 | 3.600–3.957 | 3.886 | 3.709–4.064 | 3.641 | 3.470–3.812 | 2 | 1.627 | .198 |
|  | after attack message |  | 3.588 | 3.395–3.781 | 3.598 | 3.406–3.791 | 3.535 | 3.350–3.720 |  |  |  |
| 1. ANOVA revealed a significant interaction between the impact of the attack message and the message scenarios.   CI: confidence interval; ANOVA: analysis of variance; df: degrees of freedom. | | | | | | | | | | | |
